# Combined treatment by burosumab and a calcimimetic can ameliorate hypophosphatemia due to excessive actions of FGF23 and PTH in adult XLH with tertiary hyperparathyroidism: A case report

**DOI:** 10.3389/fendo.2022.1004624

**Published:** 2022-12-02

**Authors:** Yuichi Takashi, Kyoko Toyokawa, Naoki Oda, Yoshimi Muta, Hisashi Yokomizo, Seiji Fukumoto, Daiji Kawanami

**Affiliations:** ^1^ Department of Endocrinology and Diabetes Mellitus, Fukuoka University School of Medicine, Fukuoka, Japan; ^2^ Department of Molecular Endocrinology, Fujii Memorial Institute of Medical Sciences, Institute of Advanced Medical Sciences, Tokushima University, Tokushima, Japan

**Keywords:** X-linked hypophosphatemia, tertiary hyperparathyroidism, fibroblast growth factor 23, parathyroid hormone, burosumab, evocalcet

## Abstract

**Introduction:**

X-linked hypophosphatemia (XLH) is the most prevalent type of heritable fibroblast growth factor 23 (FGF23)-related hypophosphatemic rickets. Recently, anti-FGF23 antibody, burosumab, has become clinically available. We herein report a patient with adult XLH and tertiary hyperparathyroidism.

**Case presentation:**

The serum phosphate level and tubular maximum reabsorption of phosphate per glomerular filtration rate (TmP/GFR) remained low, despite burosumab treatment. While the influence of the relationship between FGF23 and parathyroid hormone (PTH) on the phosphaturic effect is unclear, it was considered that a high level of PTH due to tertiary hyperparathyroidism remains to suppress renal phosphate reabsorption. A calcimimetic, evocalcet, increased the serum phosphate level and TmP/GFR.

**Discussion and conclusion:**

Therefore, it is important to evaluate the presence of secondary-tertiary hyperparathyroidism in patients whose serum phosphate level does not increase with burosumab treatment.

## Introduction

Rickets is a disease characterized by impaired mineralization of the growth plate and bone (1). Growth retardation and bone deformities are typical clinical features in patients with rickets. One of the important causes of rickets is chronic hypophosphatemia due to excessive actions of fibroblast growth factor 23 (FGF23), which is a bone-derived hormone that reduces serum phosphate level (2).

FGF23 binds to FGF receptor 1c (FGFR1c) and α-Klotho complex in the kidney (3), and reduces phosphate reabsorption by suppressing the expression of type IIa and IIc sodium-phosphate cotransporters in the renal proximal tubules (2). In addition, FGF23 also reduces serum 1,25-dihydroxyvitamin D [1,25(OH)_2_D] level, which enhances intestinal phosphate absorption (2). Thus, FGF23 reduces serum phosphate level by inhibiting both proximal tubular phosphate reabsorption and intestinal phosphate absorption.

While there are several types of heritable FGF23-related hypophosphatemic rickets, X-linked hypophosphatemia (XLH) is the most prevalent one (4). XLH is caused by inactivating mutations in *phosphate-regulating gene with homologies to endopeptidases on the X chromosome* (*PHEX*) with an incidence of almost 1 in 20,000 (5). XLH is a relatively not rare inherited disorder, and approximately 30-50% of patients with XLH are sporadic cases according to the previous reports (4, 6). Although how inactivating mutations in *PHEX* cause excessive production of FGF23 in the bone remains unclear, serum FGF23 levels in patients with XLH were shown to be elevated (7, 8).

Patients with XLH have been treated with oral phosphate salt and active vitamin D agents (9, 10). The goals of such therapy are to heal rickets, improve growth in children, and ameliorate osteomalacia in adults. Although this conventional therapy can improve the symptoms of rickets, at least in part, this therapy also induces some adverse effects, including secondary-tertiary hyperparathyroidism, hypercalciuria, and nephrolithiasis (11). In addition, these adverse effects can promote kidney injury, and induce the progression of chronic kidney disease (CKD). Furthermore, adherence to therapy is difficult for the patients because of the frequent dosing of phosphate salt.

Recently, anti-FGF23 antibody, burosumab, has become clinically available (12, 13). Burosumab is a fully human monoclonal IgG1 antibody against FGF23 that binds circulating active full-length FGF23, and blocks its biologic effects in target organs. This new treatment for patients with XLH is expected to ameliorate rickets, overcoming the limitations of conventional treatment.

We herein report a case of adult XLH (49-year-old female) with tertiary hyperparathyroidism under conventional treatment. First, we attempted to switch to burosumab treatment, but the serum phosphate level and tubular maximum reabsorption of phosphate per glomerular filtration rate (TmP/GFR) remained low. The high level of parathyroid hormone (PTH) due to tertiary hyperparathyroidism may have suppressed renal phosphate reabsorption despite burosumab treatment. The patient’s serum phosphate level and TmP/GFR increased with the combined treatment of burosumab and a calcimimetic, evocalcet.

## Case presentation

**Figure 1:**
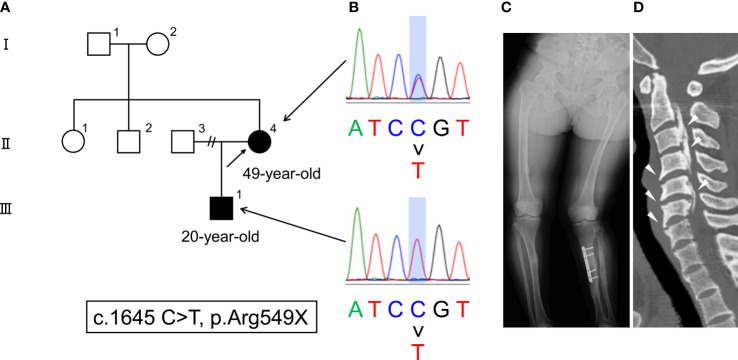
**(A)** The family pedigree of this case. The patient is described as II-4 (arrow), and her son is described as III-1. **(B)** Genetic mutations in *PHEX* (c.1645 C > T, p. Arg549X). **(C)** Radiograph of deformities in the lower extremities. **(D)** CT findings of paraspinal ligament ossification (arrowheads).

A 49-year-old female had been diagnosed with rickets at 1 year old, but the detailed pathogenesis of the rickets in this patient was unclear. She showed delayed and disproportionate growth, delayed motor development and gait abnormalities, short stature, deformity of weightbearing limbs, tooth abscesses, and excessive dental caries in childhood. Because she firstly suffered a left tibial pseudofracture, she started medication with oral phosphate salt for her hypophosphatemic rickets at 4 years old. She underwent the left tibial osteotomy for deformities of the lower extremities when she was in junior high school. Thereafter, she stopped treatment by herself during her high school days. She suffered a right hip fracture 10 years ago (the fracture was healed by conservative treatment), and restarted medication with phosphate salt without active vitamin D agents. However, despite the treatment, she repeatedly suffered pseudofractures. Furthermore, she suffered from paraspinal ligament ossification, which led to a restricted range of movement, neurological symptoms, and chronic disability, therefore, she underwent the laminoplasty of the 2nd to the 7th cervical vertebrae 8 years ago. Her son presented to a pediatrician with a chief complaint of a delayed start of walking at 1 year and 6 months old. He was also diagnosed with hypophosphatemic rickets (family pedigree is shown in **Figure 1A**). Furthermore, an elevated serum full-length FGF23 level (127 pg/mL; measured by ELISA [reference range: 16-69 pg/mL] (7)) in the son was revealed at 10 years old.

The patient was transferred to our hospital along with her son from his pediatrician. Her height was 140 cm, and her body weight was 49.0 kg (body mass index [BMI]: 25.0 kg/m^2^). While the serum phosphate level at 2 hours after taking phosphate salt (3.1 mg/dL) was maintained within the reference range (2.5-4.5 mg/dL) (the fasting serum phosphate level: 2.1 mg/dL) under conventional treatment with phosphate salt (1,700 mg/day) without active vitamin D agents, the serum full-length FGF23 level (measured by CLEIA [reference range: 19.9-52.9 pg/mL] (14) was markedly elevated (5,100 pg/mL). The TmP/GFR was low (**Table 1**). In addition, she also showed tertiary hyperparathyroidism (the serum albumin adjusted calcium level: 10.3 mg/dL, serum intact PTH level: 532 pg/mL) and CKD (the estimated GFR [eGFR]: 56.0 mL/min/1.73 m^2^) (**Table 1**). The serum levels of alkaline phosphatase (ALP), bone-type ALP (BAP), 1,25-dihydroxyvitamin D [1,25(OH)_2_D], tartrate-resistant acid phosphatase 5b (TRACP-5b), 25-hydroxyvitamin D [25(OH)D], and urinary calcium/creatinine (Cr) ratio are shown in **Table 1**. Since vitamin D deficiency was revealed, she started native vitamin D supplementation.

**Table 1:** The changes in each parameter after starting burosumab treatment.

	Reference range	Conventional treatment	Burosumab						
			1.0 mg/kg = 50 mg every four weeks						
			Evocalcet						
			without	1 mg	2 mg	4 mg	6 mg		
			Weeks after starting burosumab treatment						
			4 weeks	8 weeks	12 weeks	24 weeks	36 weeks	48 weeks	96 weeks
Pi (mg/dL) (trough)	2.5-4.5	2.1	1.4	1.3	1.8	1.5	2.6	2.4	2.5
TmP/GFR (mg/dL) (trough)	2.3-4.3	1.4			1.4	1.3	2.3	2.2	2.2
Albumin adjusted Ca (mg/dL)	8.8-10.1	10.3	10.7	10.2	9.8	10.4	8.7	8.6	8.7
Urinary Ca/Cr ratio	< 0.3	0.17	0.29	0.16	0.14	0.27	0.13	0.08	0.26
intact PTH (pg/mL)	9.3-74.9	532			463	258	144	195	154
eGFR (mL/min/1.73m^2^)	> 90	56.0	56.8	59.9	58.3	58.0	53.0	58.0	63.5
ALP (IFCC) (U/L)	38-113	135	128	133	123	143	137	129	148
BAP (μg/L)	3.7-20.9	22.9			21.0	21.2	24.1	20.9	29.1
1,25(OH)_2_D (pg/mL)	20.0-60.0	50.1			33.9	30.9	29.9	37.0	21.6
TRACP-5b (mU/dL)	170-590	615			284	678	665	596	875
25(OH)D (ng/mL)	> 20	6.7							
intact FGF23 (pg/mL)	19.9-52.9	5,100							

Pi, phosphate; TmP/GFR, tubular maximum reabsorption of phosphate per glomerular filtration rate; Ca, calcium; Cr, creatinine; PTH, parathyroid hormone; eGFR, estimated glomerular filtration rate; ALP, alkaline phosphatase; BAP, bone-type ALP; 1,25(OH)_2_D, 1,25-dihydroxyvitamin D; TRACP-5b, tartrate-resistant acid phosphatase 5b; 25(OH)D, 25-hydroxyvitamin D; FGF23, fibroblast growth factor 23.

At this stage, genetic testing for both the patient and her son revealed a previously reported mutation in *PHEX* (c.1645 C>T, p. Arg549X) (15), which led to the definitive diagnosis of XLH (**Figure 1B**). The skeletal investigation showed deformities of the lower extremities (**Figure 1C**). In addition, she suffered from paraspinal ligament ossification (**Figure 1D**). There was no pseudofracture nor fracture at the time.

**Figure 2:**
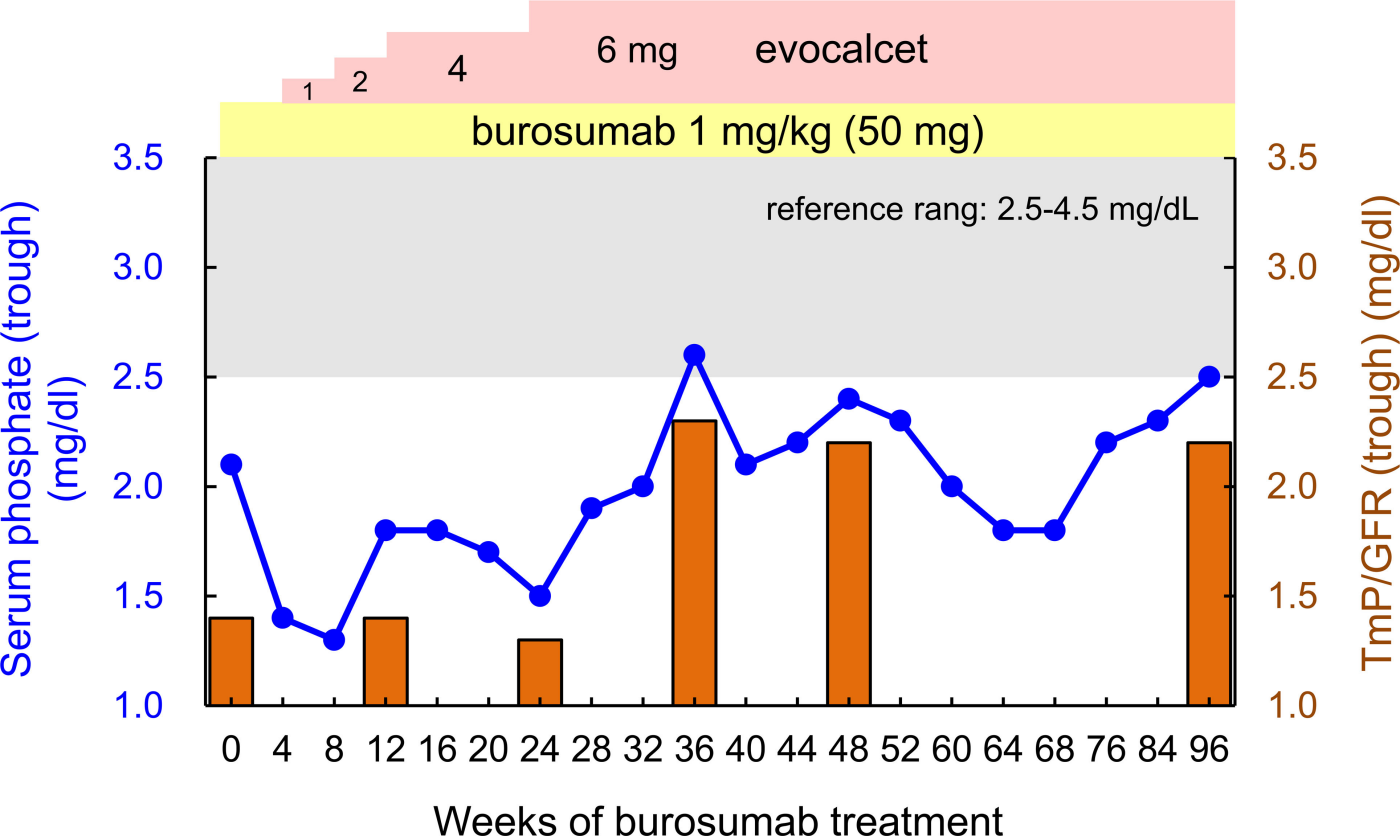
Changes in the trough (end of the dosing interval) serum phosphate levels (blue line) and TmP/GFR (orange bar) over 96 weeks of burosumab treatment. The gray zone in the panel indicates values within reference range (2.5-4.5 mg/dL), and the yellow and pink zones indicate the doses of burosumab and evocalcet, respectively.

Since we attempted to stop the progression of tertiary hyperparathyroidism and CKD, and improve her adherence to therapy, burosumab treatment was started at 1.0 mg/kg body weight (50 mg) injected subcutaneously every 4 weeks according to the approval for adult XLH by Japanese regulatory agency, and conventional treatment with phosphate salt was stopped. As shown in **Table 1** and **Figure 2**, the patient’s trough (at end of dosing interval) serum phosphate levels and TmP/GFR unexpectedly remained low even after starting burosumab treatment. While we also evaluated the peak serum phosphate level 2 weeks after administration of burosumab, she showed still hypophosphatemia (1.8 mg/dL). Although burosumab has been approved for doses of up to 2.0 mg/kg in tumor-induced osteomalacia (TIO), 1.0 mg/kg of burosumab is the maximum prescribed for adult XLH in Japan. At that time, we considered that her high level of PTH (532 pg/mL) due to tertiary hyperparathyroidism remained to suppress renal phosphate reabsorption even in the setting of burosumab treatment. Because she declined to undergo total parathyroidectomy, a calcimimetic, evocalcet, was added to inhibit PTH actions. By increasing the dose of evocalcet (1 to 6 mg/day) as shown in **Table 1** and **Figure 2**, the serum trough phosphate level started to increase 28 weeks after burosumab treatment, and the TmP/GFR also increased 36 weeks after the treatment (**Figure 2**). Furthermore, the serum albumin adjusted calcium and intact PTH levels decreased in the same period (**Table 1**). Conversely, there were no remarkable changes in the eGFR or serum levels of ALP, BAP, 1,25(OH)_2_D, TRACP-5b, or urinary calcium/Cr ratio during this period. In addition, she did not develop any injection site or general adverse events with burosumab treatment.

## Discussion and conclusion

In this case report, we described an adult XLH patient with tertiary hyperparathyroidism under conventional treatment with oral phosphate salt. Generally, patients with XLH have been treated with phosphate salt and active vitamin D agents. We consider that the treatment with phosphate salt without active vitamin D agents may have contributed to the development of tertiary hyperparathyroidism of this patient at least in part. Although we attempted to switch to burosumab treatment, controlling the serum phosphate level and TmP/GFR was difficult. The patient’s serum phosphate level and TmP/GFR ultimately increased with the combined treatment of burosumab and evocalcet.

A double-blind, placebo-controlled phase 3 trial was performed in 134 adult patients with XLH (16). Most of these patients had been treated with conventional therapy. The burosumab group received 1.0 mg/kg burosumab every 4 weeks, and administration of burosumab increased the serum phosphate level (both peak and trough), TmP/GFR, and serum 1,25(OH)_2_D level. This improvement was maintained up to 48 weeks (17). In a phase 3 trial, the serum phosphate level (both peak and trough) and TmP/GFR increased promptly within 4 weeks (16). Therefore, the effects of burosumab for adult XLH are expected to improve biochemical parameters quite rapidly. However, that trial excluded the participants likely with current case, whose serum albumin adjusted calcium level was >10.8 mg/dL and serum intact PTH level was >2.5-fold the upper limit of the reference range (16). Therefore, the efficacy of burosumab for treating adult XLH patients with tertiary hyperparathyroidism has not been elucidated.

One of the important points to discuss is whether or not PTH can suppress renal phosphate reabsorption even under burosumab treatment. The relationship between FGF23 and PTH on the phosphaturic effect is unclear at present. A recent clinical study concluded that the phosphaturic effects of FGF23 and PTH were interdependent, with both required to adequately regulate renal phosphate handling (18). The authors showed that the administration of human PTH 1-34 normalized the serum phosphate level, followed by a significant decrease in FGF23 in 11 patients with hypoparathyroidism, which is characterized by a high serum phosphate level despite concomitant FGF23 elevation (18). Furthermore, two cases of XLH with tertiary hyperparathyroidism were reported to have a normalized TmP/GFR upon being rendered hypoparathyroidism by total parathyroidectomy but still showed the marked elevation of FGF23 (19, 20). These results indicated the interdependent action on the phosphaturic effects between FGF23 and PTH.

It was also recently shown that the effect of FGF23 excess on TmP/GFR is altered by PTH, indicating that the effect is ameliorated by hypoparathyroidism and is augmented by hyperparathyroidism (21). However, those data also demonstrated that the phosphaturic effect augmented by hyperparathyroidism is altered slightly by the serum level of FGF23 (21). The authors demonstrated that the effect of FGF23 on TmP/GFR is significant at the 16th percentile and at the 50th percentile of PTH, but not at the 84th percentile of PTH (21). Thus, we consider that blockade of FGF23 action by burosumab does not increase TmP/GFR under conditions of a high PTH level. Incidentally, the present case demonstrates the phosphaturic effect of PTH under blockade of FGF23 by burosumab in an adult case of XLH. Although several reports have described the effects of calcimimetics for XLH under conventional treatment (22, 23), this is the first to demonstrate the possible effect of combined treatment with burosumab and calcimimetics on regulating the serum phosphate level and TmP/GFR in FGF23-related hypophosphatemic rickets/osteomalacia, including XLH and TIO. Although there are several limitations for parathyroid surgery such as the risk of surgery itself, postoperative hypothyroidism, and recurrence of hyperparathyroidism, the treatment with calcimimetics can avoid these disadvantages. In addition, some patients cannot undergo the surgery because of rejection of surgery or religious objections. On the other hand, it is unclear whether calcimimetics can control tertiary hyperparathyroidism in all patients. Despite the activation of calcium-sensing receptor by calcimimetics potentially increase urinary calcium excretion (24), urinary calcium/Cr ratio of the patient was not changed after administration of evocalcet (**Table 1**).

We are aware of the criticism that burosumab was unable to inhibit the FGF23 action completely in this case because the patient’s serum FGF23 level was markedly elevated. Burosumab has been approved for FGF23-related hypophosphatemic rickets/osteomalacia, including XLH and TIO, in several countries, and is expected to be effective in preventing complications due to conventional therapy, as shown above. However, it was recently claimed that the effects of burosumab on the clinical outcomes of TIO were not significant or even moderate compared with its efficacy in cases of XLH (24). For instance, the tubular reabsorption of phosphate remained low, the mean serum phosphate level was barely in the reference range, most fractures persisted after 2 years of the treatment, and new fractures continued to appear. It was suggested that a contributor to the low response in the TIO studies relative to the findings in the XLH study was because patients with TIO had more severe disease than those with XLH, as the baseline serum phosphate level was 1.6 mg/dL (TIO) versus 1.9 mg/dL (XLH), and the baseline FGF23 levels were high at 416 pg/mL to 1018 pg/mL (TIO) (16, 25–27). Even though the present case involved XLH, it is possible that 1.0 mg/kg of burosumab for FGF23-related hypophosphatemic rickets/osteomalacia with a high serum FGF23 level was not enough to inhibit the FGF23 action completely, regardless of XLH or TIO.

In conclusion, while the effect of the relationship between FGF23 and PTH on the phosphaturic activity is controversial, it is considered that the high level of PTH due to tertiary hyperparathyroidism remains to suppress renal phosphate reabsorption in the present case. Since a calcimimetic, evocalcet, increased the serum phosphate level and TmP/GFR, it is important to evaluate the presence of secondary-tertiary hyperparathyroidism in patients whose serum phosphate level does not increase with burosumab treatment, and to consider the addition of calcimimetics for such treatment. Because patients with adult XLH and secondary-tertiary hyperparathyroidism, such as the current case, have been excluded from clinical trials of burosumab, further reports or studies are required to evaluate the possible effect of burosumab on XLH with secondary-tertiary hyperparathyroidism, and its long-term effects need to be assessed as well.

## Data availability statement

The original contributions presented in the study are included in the article/Supplementary Material. Further inquiries can be directed to the corresponding author.

## Ethics statement

The studies involving human participants were reviewed and approved by The medical ethics committee of Fukuoka University Hospital (July 28, 2020). The patients/participants provided their written informed consent to participate in this study.

## Author contributions

YT was involved in conceptualization, data curation, writing and editing the manuscript. KT performed the mutation analysis for *PHEX* gene. NO, YM and HY performed the clinical investigations. SF reviewed the manuscript. DK supervised this study and reviewed the manuscript. All authors contributed to the article and approved the submitted version.

## Acknowledgments

We thank all members taking care of the patient in the Department of Endocrinology and Diabetes Mellitus, Fukuoka University Hospital.

## Conflict of interest

The authors declare that the research was conducted in the absence of any commercial or financial relationships that could be construed as a potential conflict of interest.

## Publisher’s note

All claims expressed in this article are solely those of the authors and do not necessarily represent those of their affiliated organizations, or those of the publisher, the editors and the reviewers. Any product that may be evaluated in this article, or claim that may be made by its manufacturer, is not guaranteed or endorsed by the publisher.
